# 

**Published:** 1908-04-11

**Authors:** 


					April 11, 1908. THE HOSPITAL.
CONTENTS.
SomBHnSpital Conference, 1908
27
Soma a vumerence,
Annnr*.!?"ts of Criminal
28
Annotations-
^ ui nit; unmmai
Enzymes in Bananas?Insurance against Actions for Negli-
TVI?<i?en<ie~^)ay Nurseries
29
fvrA*t? nurseries
nn 'P."1, Opluipn and Movement
30
Hospital Clinics?
?1UU i-TJLUVcZUt*U L
^ (TTlli^a^^es Children in Disease.
By David Forsyth, M.D.,
M.R.c I>.
33
Special Article-
Perineal Prostatectomy
XVTarit ~
34
Medicine?
T?pU?.???"la in Typhoid Fever
37
38
Poiw^in.Sur&ery-
Aneurys
39
Some Aspects of General Peritoniti
T.a
40
laTh?KoloS:y and Rhinology
rni  mimoiogy-
-Wie Operation for Adenoid
Dermatolog-y-
42
Scabies in General Practice
42
Obstetrics ?  M?n?rompnt, of the Breasts ... 43
Cracked Nipples " and the Management of the Breasts
43
Therapeutics -
t> nnH Disnonsmff
Prescriptions and Dispensing ...
- ??  n<.3?t<tlnnf>r'!l (
Column?
44
The General Practitioner's Column
within the Abdomen   45
Arresthetisation for Operations within the Abdomen
a : *-* ---1 nffinoos' 1li-nartm?nt?
45
Resident Medical Officers' Department-
"?"l. ? .nffimrs tiTirl tlio i\ursmtr Start   4b
The Resident Medical Officers and the Nursing Staff
Hospital ad ministration-
TTnsnitn.ls Conference, 1908  47
The United Kingdom Hospitals Conference, 1908 ..
47
News and Coming Events
51
New. Appliances and TMngf Mescal -
52
A Machine ""inducing Artificial Respiration
52
Iffursing- Administration
53
The Uniform Certificate?II.
53
Editor's letter-Box?   54
Bier's Treatment or Unna's Paste
54

				

